# Exploring intrinsic variability between cultured nasal and bronchial epithelia in cystic fibrosis

**DOI:** 10.1038/s41598-023-45201-4

**Published:** 2023-10-30

**Authors:** Lisa W. Rodenburg, Mieke Metzemaekers, Isabelle S. van der Windt, Shannon M. A. Smits, Loes A. den Hertog-Oosterhoff, Evelien Kruisselbrink, Jesse E. Brunsveld, Sabine Michel, Karin M. de Winter-de Groot, Cornelis K. van der Ent, Ralph Stadhouders, Jeffrey M. Beekman, Gimano D. Amatngalim

**Affiliations:** 1 Department of Pediatric Pulmonology, Wilhelmina Children’s Hospital, University Medical Center Utrecht, Utrecht University, Member of ERN-LUNG, 3584 EA Utrecht, The Netherlands; 2 Regenerative Medicine Center Utrecht, University Medical Center Utrecht, Utrecht University, 3584 CT Utrecht, The Netherlands; 3 Department of Pulmonary Medicine, Erasmus University Medical Center, 3015 CE Rotterdam, The Netherlands; 4 Department of Cell Biology, Erasmus University Medical Center, 3015 CE Rotterdam, The Netherlands; 5 Centre for Living Technologies, Alliance TU/e, WUR, UU, UMC Utrecht, 3584 CB Utrecht, The Netherlands

**Keywords:** Cystic fibrosis, Cell biology

## Abstract

The nasal and bronchial epithelium are unified parts of the respiratory tract that are affected in the monogenic disorder cystic fibrosis (CF). Recent studies have uncovered that nasal and bronchial tissues exhibit intrinsic variability, including differences in mucociliary cell composition and expression of unique transcriptional regulatory proteins which relate to germ layer origin. In the present study, we explored whether intrinsic differences between nasal and bronchial epithelial cells persist in cell cultures and affect epithelial cell functioning in CF. Comparison of air–liquid interface (ALI) differentiated epithelial cells from subjects with CF revealed distinct mucociliary differentiation states of nasal and bronchial cultures. Moreover, using RNA sequencing we identified cell type-specific signature transcription factors in differentiated nasal and bronchial epithelial cells, some of which were already poised for expression in basal progenitor cells as evidenced by ATAC sequencing. Analysis of differentiated nasal and bronchial epithelial 3D organoids revealed distinct capacities for fluid secretion, which was linked to differences in ciliated cell differentiation. In conclusion, we show that unique phenotypical and functional features of nasal and bronchial epithelial cells persist in cell culture models, which can be further used to investigate the effects of tissue-specific features on upper and lower respiratory disease development in CF.

## Introduction

The nasal and bronchial airway epithelium are unified parts of the human respiratory tract that originate from ecto- and endodermal germ layers, respectively^1,2^. Despite differences in embryonic origin, both nasal and bronchial epithelial layers display a pseudostratified morphology and consist of ciliated, secretory and basal cells^3^. Moreover, nasal and bronchial epithelial cells employ common mechanisms to provide respiratory host defence^4–6^.

Nasal and bronchial epithelial cells are furthermore mutually affected in multiple respiratory diseases, including the monogenic disorder cystic fibrosis (CF)^7^. CF is caused by autosomal recessive inherited mutations in the cystic fibrosis transmembrane conductance regulator (*CFTR*) gene^8^. These genetic defects attenuate CFTR protein-dependent chloride conductance and fluid secretion at the surface of the airway epithelium^9^. This results in accumulation of dehydrated mucus at the epithelial surface, which cannot be removed via mucociliary clearance and consequently leads to the development of a muco-obstructive respiratory disease^10^.

In vitro models are widely used to study impaired airway epithelial cell functions in CF and the efficacy of novel CFTR-modulating therapies. These models commonly use undifferentiated airway basal progenitor cells from nasal or bronchial tissues^11^. Air–liquid interface (ALI) differentiated airway epithelial cell cultures are regarded as the golden standard^11^, although airway organoids are emerging as a novel advanced 3D model system, uniquely suited for investigating fluid secretion^12,13^.

Recent transcriptome studies with native airway tissue samples have uncovered intrinsic differences between the nasal and bronchial epithelium, including variations in ciliated and secretory cell composition and the expression of unique transcriptional regulatory proteins that relate to a difference in germ layer origin^14–17^. It remains unexplored whether these tissue-specific hallmarks affect nasal and bronchial epithelial cell functioning, and therefore have differential outcomes on upper and lower respiratory disease development in CF.

In the present study, we determined whether in vitro models can be used to explore the role of unique nasal and bronchial characteristics on airway epithelial cell functioning in CF. First, we compared paired nasal and bronchial epithelial cells from individuals with CF that were differentiated in ALI-cultures, and used RNA sequencing (RNA-seq) to determine persistent differences in epithelial differentiation and the expression of unique transcriptional regulatory proteins. Next, we performed an assay for transposase-accessible chromatin using sequencing (ATAC-seq) to determine whether differences in transcriptional regulatory proteins are epigenetically imprinted already in basal progenitor cells. Moreover, we examined epithelial fluid secretion in nasal and bronchial organoids in a forskolin-induced swelling (FIS) assay, and investigated how CFTR-independent fluid secretion is affected by increased ciliated cell differentiation in CF nasal organoids.

## Results

### Nasal and bronchial epithelial cell cultures exhibit a unique mucociliary differentiation state and transcriptome

**Figure 1:**
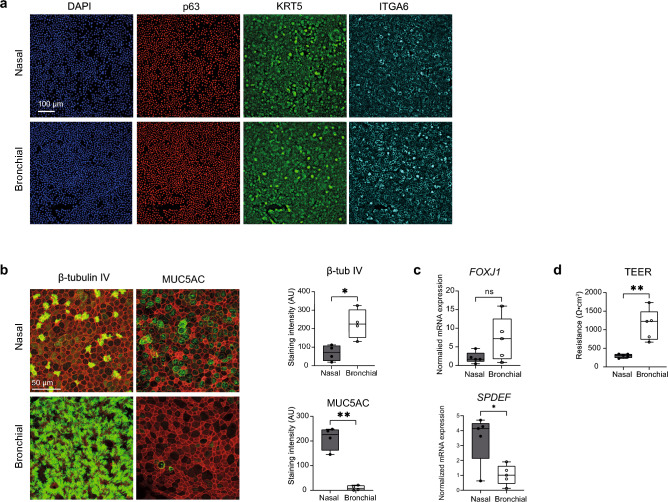
Nasal and bronchial epithelial cell cultures from individuals with CF exhibit unique mucociliary differentiation states. (**a**) Representative IF staining of the basal cell markers p63 (red), KRT5 (green), and ITGA6 (cyan) in undifferentiated CF nasal and bronchial epithelial cells (F508del/F508del). DAPI (blue) was used to stain nuclei. Scale bar equals 100 µm. (**b**) Representative IF staining (left panel) and quantification (right panel) of β-tubulin IV (ciliated cells) and MUC5AC (goblet cells) in paired ALI-differentiated nasal and bronchial cells of CF subjects (n = 4 independent donors; all F508del/F508del). Cultures were differentiated for 18 days. Epithelial markers are shown in green, phalloidin (red) was used as actin cytoskeleton staining. Scale bar equals 50 µm. For quantification, 3 microscopic fields were analysed per well. (**c**) mRNA expression of the cell type-specific transcriptional factors *FOXJ1* (ciliated cells) and *SPDEF* (goblet cells) in paired ALI-differentiated nasal and bronchial epithelial cells of CF subjects (n = 5 independent donors; F508del/F508del, F508del/F508del, F508del/A455E, F508del/A455E, F508del/1717-1G>A). (**d**) TEER measurements of paired ALI-differentiated nasal and bronchial epithelial cells of CF subjects (n = 5 independent donors; F508del/F508del, F508del/F508del, F508del/A455E, F508del/A455E, F508del/1717-1G>A). Data is shown as mean ± SD. Analysis of differences was conducted using paired *t*-tests (**b**–**d**). *ns* non-significant, *p < 0.05, **p < 0.01.

**Figure 2:**
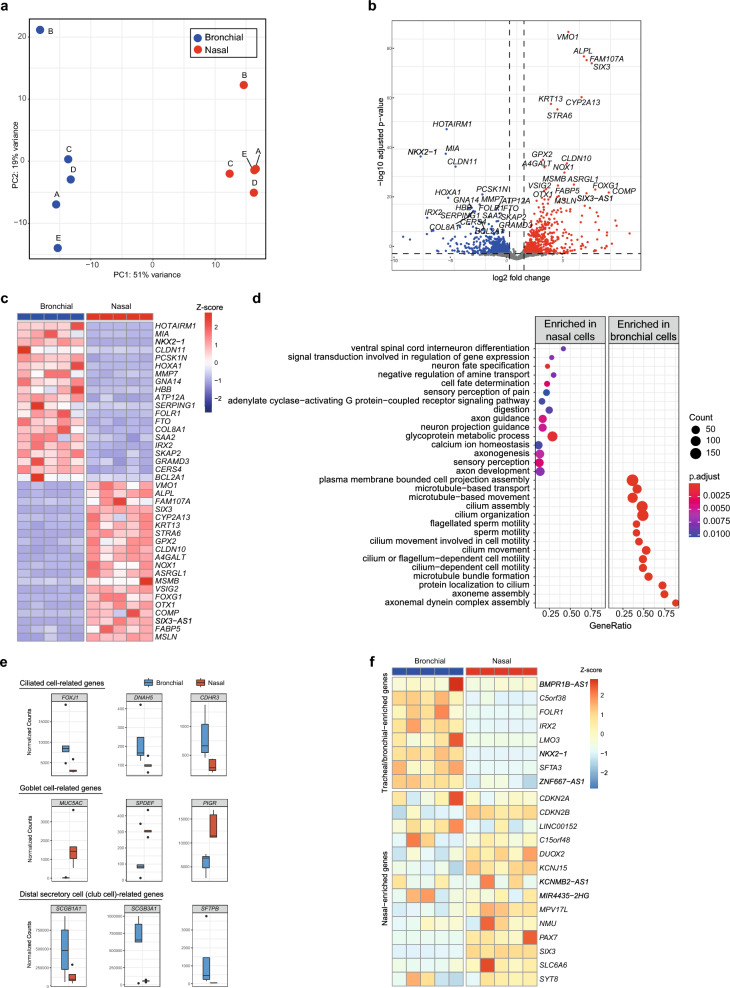
Transcriptome analysis of nasal and bronchial epithelial cells reveals unique core gene signatures. (**a**) PCA of transcriptomic analysis of paired ALI-differentiated nasal (red) and bronchial (blue) cells of CF subjects (n = 5 independent donors; F508del/F508del, F508del/F508del, F508del/A455E, F508del/A455E, F508del/1717-1G>A). A–E indicate individual donors. (**b**) Volcano plot showing DEGs (defined as adjusted p value < 0.01 and fold change > 1.5) between paired ALI-differentiated nasal and bronchial epithelial cells of CF subjects (n = 5 independent donors; F508del/F508del, F508del/F508del, F508del/A455E, F508del/A455E, F508del/1717-1G>A). Red dots indicate enriched genes in nasal epithelial cells and blue dots indicate enriched genes in bronchial epithelial cells. (**c**) Heatmap showing the 20 most significantly enriched genes in nasal (red) and bronchial (blue) epithelial cells. Z scores of normalized expression values are depicted. (**d**) Gene set enrichment analysis showing the top 15 of enriched GO terms in ALI-differentiated nasal and bronchial epithelial cells (n = 5 independent donors; F508del/F508del, F508del/F508del, F508del/A455E, F508del/A455E, F508del/1717-1G>A). Color indicates the degree of significance and dot size indicates gene count. Gene ratio explains the fraction of DEG’s in the specific GO term. (**e**) Normalized counts of ciliated cell-related genes (upper panel), goblet cell-related genes (middle planel) and distal airway secretory cell (club cell)-related genes (lower panel) in paired ALI-differentiated nasal (red) and bronchial (blue) cells of CF subjects (n = 5 independent donors; F508del/F508del, F508del/F508del, F508del/A455E, F508del/A455E, F508del/1717-1G>A). (**f**) Heatmap showing gene expression in paired ALI-differentiated nasal (red) and bronchial (blue) cells of CF subjects (n = 5 independent donors; F508del/F508del, F508del/F508del, F508del/A455E, F508del/A455E, F508del/1717-1G>A) of nasal- and trachea/bronchial-enriched genes based on scRNA-seq by Deprez et al.^15^.

Previous studies have revealed important variations in mucociliary differentiation states of freshly harvested nasal versus bronchial epithelial tissues^15^. These observations prompted us to uncover the phenotypes of cultured nasal and bronchial epithelial cells form paediatric individuals with CF. We isolated and expanded nasal and bronchial epithelial cells and confirmed their identity by immunofluorescence (IF) staining based on protein expression of basal progenitor cell markers (Fig. 1a). Paired nasal and bronchial epithelial cells were subsequently differentiated in an ALI model using similar culture conditions^18^. Differentiated nasal cell cultures contained a significantly higher percentage of MUC5AC^+^ goblet cells compared to differentiated bronchial cell cultures, which were enriched for β-tubulin IV^+^ ciliated cells (Fig. 1b). In contrast to differences in MUC5AC^+^ goblet cells, we observed CC10^+^ secretory club (-like) cells in both nasal and bronchial cell cultures (Fig. S1a). Furthermore, despite the lack of goblet cells in bronchial cell cultures in intrinsic culture conditions, we were able to induce goblet cell differentiation with the Th2 cytokine IL-13 (Fig. S1b). In line with IF staining, differentiated nasal and bronchial cells displayed higher mRNA expression of the transcription factors (TF) SPDEF and FOXJ1, respectively (Fig. 1c). In addition to a discrepancy in mucociliary cell composition, ALI-differentiated nasal cells displayed a significantly lower trans-electrical epithelial resistance (TEER) compared to cultured bronchial cells (Fig. 1d). Similar differences in mucociliary differentiation and TEER were observed in unpaired nasal and bronchial cultures from non-CF subjects (Supplementary Fig. S1c–e). Next, we profiled the transcriptomes of ALI-differentiated nasal and bronchial cultures from five individuals with CF by RNA-seq. Principal component analysis (PCA) showed clear clustering based on nasal or bronchial origin (Fig. 2a). We identified 919 and 735 differentially expressed genes (DEGs) that were higher expressed in differentiated bronchial and nasal cells, respectively (foldchange > 1.5 and adjusted p < 0.01) (Fig. 2b,c, Supplementary Table S1). Gene set enrichment analysis of these DEGs revealed higher expression of cilia-related gene sets in bronchial cells, while nasal cells showed enrichment of genes involved in neural development-related processes (Fig. 2d). In addition to enhanced expression of genes associated with ciliated cells, bronchial cells displayed higher expression of distal airway club cell-related genes (Fig. 2e). In contrast, nasal cells more abundantly expressed goblet cell-related genes (Fig. 2e). We further conducted a comparison of mRNA expression levels of adherens and tight junction-associated genes to elucidate the variations in TEER between nasal and bronchial cell cultures (Supplementary Fig. S1f). Indeed, supporting a lower barrier integrity in nasal cell cultures, we observed lower expression of ZO-1 (TJP1), E-cadherin (CDH1) and several tight junction-associated claudins. We then determined the expression of gene panels that were previously found to be specifically enriched in either nasal or tracheal/bronchial epithelial cells^15^. All of the known bronchial-enriched genes were indeed higher expressed in bronchial cell cultures compared to nasal cell cultures. Cultured nasal cells showed elevated expression of 8/14 of the reported nasal-specific genes (Fig. 2f)^15^. Overall, these results support the notion that nasal and bronchial epithelial cells exhibit unique cell type-specific differentiation characteristics that persists in cell culture.

### Identification of nasal and bronchial cell-specific signature TFs and epigenomic features

**Figure 3:**
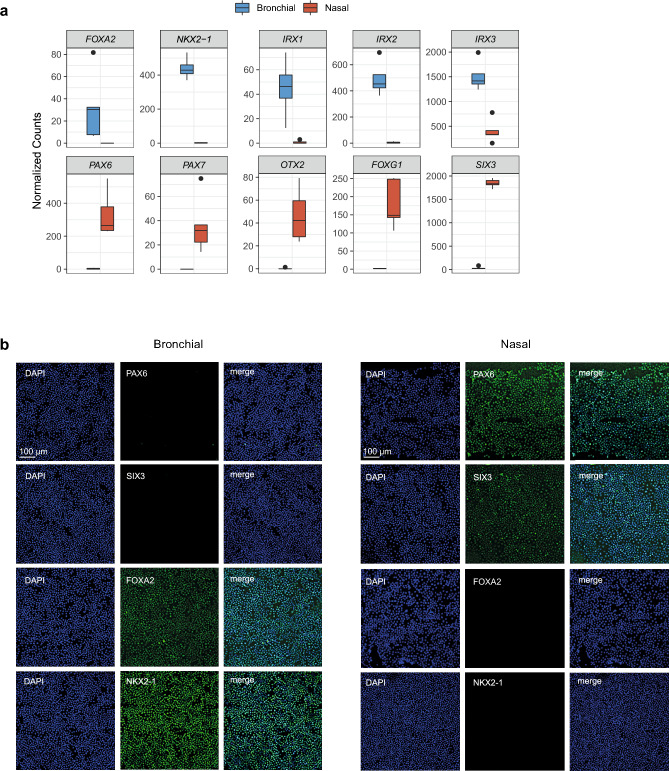
Identification of nasal and bronchial cell-specific signature TFs. (**a**) Normalized counts of RNA-seq analysis from nasal- and bronchial-specific TFs in paired ALI-differentiated nasal (red) and bronchial (blue) cells of CF subjects (n = 5 independent donors; F508del/F508del, F508del/F508del, F508del/A455E, F508del/A455E, F508del/1717-1G>A). (**b**) Representative IF staining of paired undifferentiated nasal and bronchial epithelial cells of an individual with CF (F508del/F508del). Cells were stained for the bronchial epithelial markers NKX2-1 and FOXA2 and the nasal epithelial markers PAX6 and SIX3. Scale bar equals 100 µm.

**Figure 4:**
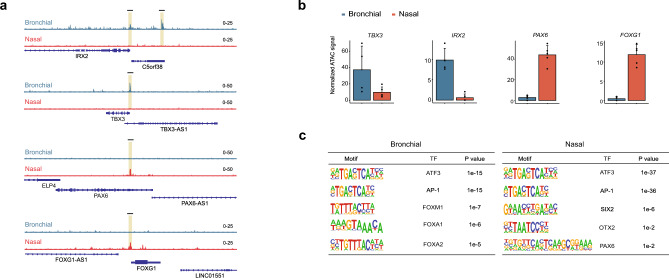
Unique epigenomic features of nasal and bronchial basal progenitor cells. (**a**) Representative genome browser shots of ATAC-seq peaks across the genes *IRX2, TBX3, PAX6* and *FOXG1*. Five paired nasal and bronchial cell cultures were analyzed (4 donors carrying the F508del/F508del mutation, 1 donor F508del/A455E). One bronchial sample was omitted for further analysis due to insufficient sample quality. (**b**) Quantified ATAC-seq signals of selected genes in the different donors (n = 4–5 independent donors). (**c**) Selection of enriched motif matrices and corresponding TF in nasal and bronchial epithelial cells, predicted by the HOMER motif analysis. Data is shown as mean ± SD.

We employed our RNA-seq dataset to unravel whether specific TFs are putative regulators of differences in differentiation between nasal and bronchial epithelial cells. We searched for TFs among the top enriched DEGs in bronchial cells and identified the TFs *FOXA2*, *NKX2-1* and *IRX1-3,* which are all involved in lung endoderm morphogenesis (Fig. 3a, upper panel)^19–23^. In contrast, top DEGs in nasal cells consisted of several unique TFs which are implicated in neural ectoderm development. These included *PAX6* and *PAX7*^24–26^, *OTX2*^27,28^, *FOXG*^29^ and *SIX3*^30^ (Fig. 3a, lower panel). Differential expression of a subset of bronchial- and nasal-specific TFs was validated at the protein level in undifferentiated basal progenitor cells (Fig. 3b). We next performed ATAC-seq to elucidate whether nasal and bronchial tissue-specific signature TFs and their cognate DNA binding sites were associated with accessible chromatin in basal progenitor cells. Of the five paired nasal and bronchial cell cultures, one bronchial sample was omitted for further analysis due to insufficient sample quality. We reproducibly detected a total of 15.434 peaks of chromatin accessibility across samples, of which 147 regions were significantly more accessible and specific for nasal and 58 for bronchial cells (log2 fold change > 1 and adjusted p < 0.1) (Supplementary Fig. S2a,b, Supplementary Table S2). Pathway enrichment analysis of genes associated with nasal- or bronchial-specific accessible regions revealed distinct sets of biological processes (Supplementary Fig. S2c). Examples of individual genes near ATAC-seq peaks that were specifically enriched in bronchial cells include *IRX2* and *TBX3*, both involved in early lung development (Fig. 4a,b)^31,32^. In accordance with mRNA expression in differentiated ALI-cultures, genes linked to nasal-specific regions of accessible chromatin included *PAX6* and *FOXG1* (Fig. 4a,b)^24,29^. Integration of RNA-seq results from ALI-differentiated bronchial cells and ATAC-seq data from basal progenitor bronchial cells yielded twelve overlapping genes for bronchial cells, including *IRX1* and *IRX2* (Supplementary Fig. S2d, left panel)*.* For nasal cells, 19 overlapping genes were found, including the signature TFs *PAX6* and *FOXG1* (Supplementary Fig. S2d, right panel). A TF motif enrichment analysis was conducted to search for known TF-binding motifs in accessible regions. Both bronchial- and nasal-specific regulatory regions were enriched for binding motifs of the stress-induced TF ATF-3^33^ and AP-1, which regulates cellular functions including proliferation, differentiation and apoptosis^34^ (Fig. 4c). Binding motifs for FOXM1, which has been implicated in several lung diseases^35^ as well as those for FOXA1 and FOXA2, both involved in lung morphogenesis^19^, were significantly enriched in bronchial cells. Nasal-specific enriched binding motifs were found for PAX6, SIX2 and OTX2. Altogether, we found unique nasal- and bronchial specific TF, which in part are imprinted epigenetically in airway basal progenitor cells.

### ALI culture-derived nasal and bronchial organoids display distinctive fluid secretion

**Figure 5:**
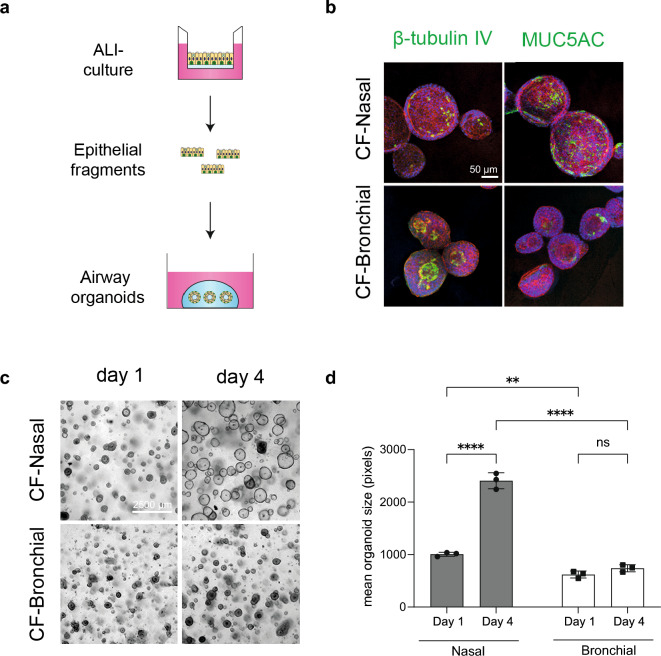
ALI culture-derived nasal and bronchial organoids display distinctive morphological features. (**a**) Schematic representation showing how ALI-differentiated airway epithelia are converted into organoids to study organoid size and FIS. (**b**) Representative IF staining of paired nasal and bronchial airway organoids of a CF subject (F508del/2183AA>G), showing β-tubulin IV (ciliated cells) and MUC5AC (goblet cells) staining (in green). Phalloidin (red) was used as actin cytoskeleton staining and DAPI (blue) to stain nuclei. Scale bar equals 50 µm. (**c**) Representative brightfield images of paired CF nasal and bronchial airway organoids (F508del/F508del) at day 1 and 4 after plating of epithelial fragments, showing lumen formation in nasal but not in bronchial organoids. Scale bar equals 2500 µm. (**d**) Quantification of mean organoid size (pixels) of paired CF nasal and bronchial airway organoids at day 1 and 4 after plating of epithelial fragments (n = 3 wells from 1 donor; F508del/F508del). Data is shown as mean ± SD. Analysis of differences was conducted with a two-way ANOVA with Tukey post-hoc test (**d**). *ns* non-significant, **p < 0.01, ****p < 0.0001.

**Figure 6:**
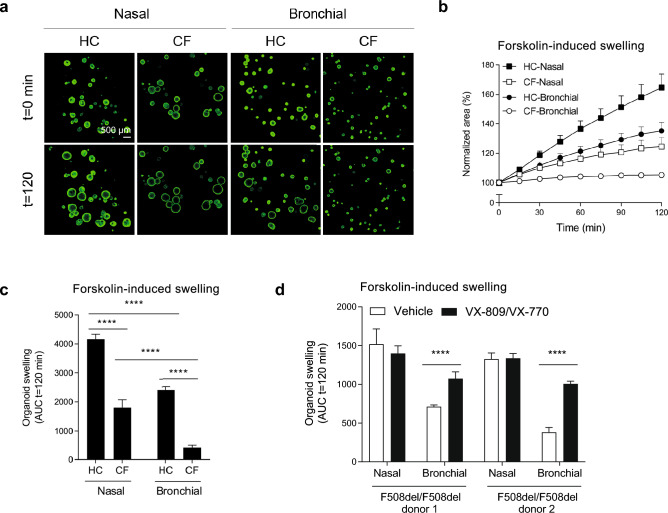
Differences in CFTR-independent forskolin-induced fluid secretion between nasal and bronchial organoids. (**a**) Representative confocal images of calcein green-stained nasal and bronchial airway organoids from HC and CF (F508del/2183AA>G) at t = 0 and t = 120 min after stimulation with forskolin (5 µM). Scale bar equals 500 μm. (**b**) Nasal and bronchial airway organoids from HC (n = 3 independent donors) and CF (n = 3 independent donors; F508del/F508del, F508del/F508del, F508del/1717-1G>A) subjects were stimulated with forskolin (5 µM) and organoid swelling was measured in time, demonstrating differences in fluid secretion. Results are depicted as percentage increase in normalized area in time. (**c**) Nasal and bronchial airway organoids from HC (n = 3 independent donors) and CF (n = 3 independent donors; F508del/F508del, F508del/F508del, F508del/1717-1G>A) subjects were stimulated with forskolin (5 µM) and organoid swelling was measured in time, demonstrating differences in fluid secretion. Results are depicted as area under the curve (AUC) plots (t = 120 min). (**d**) Paired nasal and bronchial airway organoids from CF subjects with a F508del/F508del genotype (n = 2 independent donors) were pre-treated with VX-809 (10 µM) for 48 h, and subsequently acute stimulated with forskolin (5 µM) together with VX-770 (10 µM) or vehicle. Airway organoid swelling is depicted as AUC plots (t = 120 min). Data is shown as mean ± SD. Swelling assays were conducted in quadruplicates for each condition. Analysis of differences was conducted with a two-way ANOVA with Bonferroni post-hoc test (**c**,**d**). ****p < 0.0001.

**Figure 7:**
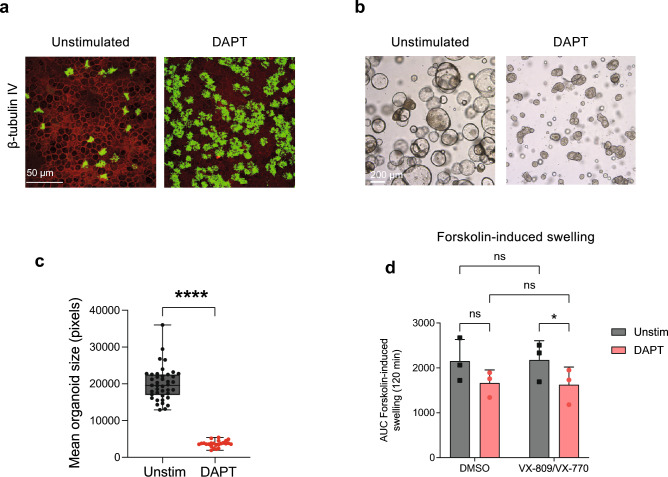
Intrinsic fluid secretion in nasal organoids is reduced by ciliated cell enrichment. (**a**) Representative IF staining of β-tubulin IV (ciliated cells) in nasal cells cultured with or without the γ-secretase inhibitor DAPT (20 µM) from an individual with CF (F508del/F508del). Cultures were differentiated for 18 days. Epithelial markers are shown in green, phalloidin (red) was used as actin cytoskeleton staining. Scale bar equals 50 µm. (**b**) Representative brightfield images of nasal organoids treated with or without the γ-secretase inhibitor DAPT (20 µM), from an individual with CF (F508del/F508del), 6 days after plating. Scale bar equals 200 µm. (**c**) Quantification of mean organoid size of nasal organoids treated with or without the γ-secretase inhibitor DAPT (20 µM) from an individual with CF (F508del/F508del) (n = 24/41 wells). (**d**) Nasal organoid swelling assay with organoids from individuals with CF (n = 3 independent donors; all F508del/F508del), treated with or without the γ-secretase inhibitor DAPT (20 µM). Organoids were stimulated with forskolin (5 µM) alone, or with forskolin (5 µM) together with VX-770 (10 µM) and pre-treatment with VX-809 (10 µM) for 48 h. Results are depicted as AUC plots (t = 120 min). Data is shown as mean ± SD. Swelling assays were conducted in quadruplicates for each condition. Analysis of differences was conducted with an unpaired *t*-test (**c**) or two-way ANOVA with Tukey post-hoc test (**d**). *ns* non-significant, *p < 0.05, ****p < 0.0001.

Next, we determined whether differences in mucociliary differentiation between ALI-cultured nasal and bronchial epithelial cells affect fluid secretion. We employed 3D airway organoids generated from ALI-differentiated epithelial fragments (Fig. 5a)^18^. In line with our previous observations in ALI-cultures (Fig. 1), MUC5AC^+^ and β-tubulin IV^+^ cells were more abundant in CF nasal and bronchial organoids, respectively (Fig. 5b). Evaluation of organoid morphology revealed that nasal epithelial sheets formed into cystic organoids with clearly identifiable lumens, compared to CF donor-matched bronchial organoids that had limited fluid secretion leading to a small lumen size (Fig. 5c,d). Similar differences between nasal and bronchial organoid morphology were observed in cultures derived from healthy control (HC) subjects (Supplementary Fig. S3a). This suggests differences in intrinsic fluid secretion between nasal and bronchial organoids, presumably independent of CFTR under basal culture conditions. To further investigate fluid secretion properties, we measured organoid swelling in response to the cAMP agonist forskolin, which we previously identified as an inducer of CFTR-independent fluid secretion in CF nasal organoids^13,18^. Nasal and bronchial organoids from HC subjects (unpaired samples) were included in this FIS assay, to discriminate between CFTR-dependent and CFTR-independent swelling responses. We observed more prominent swelling responses in nasal and bronchial organoids from HC subjects compared to those from individuals with CF, which can be explained by a dysfunctional cAMP-dependent CFTR channel in CF organoids (Fig. 6a–c). Remarkably, nasal organoids from CF subjects displayed a significantly higher FIS when compared to paired bronchial organoids, which is likely CFTR-independent. Furthermore, bronchial organoids from subjects with CF with a F508del homozygous genotype displayed enhanced FIS in response to the CFTR-repairing drugs VX-809/VX-770 (Fig. 6d). Paired nasal organoids did not respond to these drugs, also probably due to abundant CFTR-independent organoid swelling. To determine whether differences in fluid secretion between nasal and bronchial organoids were related to the differential expression of ion and fluid secretion channels and transporters, we conducted a further analysis of our RNA sequencing data (Supplementary Fig. S3b). We did not observe significant differences in the expression of apical ion channels and transporters, including *CFTR*, *ANO1*, *SLC26A9*, and *SLC26A4*. However, nasal cells displayed higher expression of aquaporin 3 and 5 (*AQP3* and *AQP5*), which potentially are involved in the increased fluid secretion observed in nasal organoids. Furthermore, nasal cells exhibited elevated expression of sodium transporter (ENaC) subunits, which would suggest more abundant Na^+^ and fluid absorption. Moreover, nasal cells express higher expression of several basolateral anion exchangers, including the electrogenic sodium bicarbonate cotransporter NBC (*SLC4A4*), the Na–K–Cl cotransporter NKCC1 (*SLC12A2*), the ATPase Na^+^/K^+^ transporting subunit alpha 1 (*ATP1A1*), basolateral Na^+^/H^+^ exchanger *SLC9A1*, and K^+^ channels (*KCNJ15* and *KCNQ1*). These findings demonstrate the complex and multifaceted nature of the differences in nasal and bronchial fluid secretion and absorption, which may depend on interactions among differentially expressed channels and transporters. Next, we aimed to decipher whether the observed differences in differentiation state and capacity for fluid secretion between nasal and bronchial organoids had a causative relationship. To address this issue, we employed the γ-secretase inhibitor DAPT—an inhibitor of Notch signaling—to enrich for ciliated cells in nasal organoids^36,37^. Microscopic evaluation showed the successful differentiation of DAPT-treated samples as evidenced by an increased number of β-tubulin IV^+^ ciliated cells in ALI-differentiated nasal cell cultures of CF subjects (Fig. 7a). This was confirmed at the mRNA level, showing enhanced and reduced expression of *FOXJ1* (ciliated cells) and *MUC5AC* (goblet cells), respectively (Supplementary Fig. S3c). After conversion of ALI cultures into organoids, we observed that differentiation with DAPT attenuated organoid lumen formation, suggesting reduced epithelial fluid secretion upon ciliated cell enrichment (Fig. 7b,c). However, FIS and responses to CFTR-repairing drugs were only minimally changed in CF nasal cultures differentiated with DAPT (Fig. 7d, Supplementary Fig. S3d). Altogether, the distinct swelling responses of nasal and bronchial organoids suggest that their unique differentiation states and morphological characteristics coincide with intrinsic differences in their potency to secrete fluid in a CFTR-independent manner.

## Discussion

This study aimed to explore whether cultured nasal and bronchial epithelia can be used to investigate imprinted tissue-specific characteristics in CF. Upon cultivation under similar culturing conditions, ALI-differentiated nasal and bronchial cell cultures displayed significant enrichment for goblet and ciliated cells respectively. These observations are in line with previously published single cell and bulk RNA-seq studies on freshly isolated nasal and tracheal or lower airway cells^14–17^. Therefore, we demonstrate that tissue-specific differences are preserved in cell cultures with our culture media after multiple weeks of culturing. The unique differentiation characteristics of nasal and bronchial cells appear to be independent of CF disease since similar observations were made for cells from healthy donors.

Unlike the exploration of differences between nasal and bronchial epithelial cells concerning secretory and ciliated cells, we did not investigate variations in rare airway epithelial cell populations, such as ionocytes. This highlights the need for further studies in this area. Furthermore, it is important to note that our observations were made using in-house developed expansion and differentiation medium conditions^38^. Indeed, it has been shown that the method of expanding airway basal cells may impact subsequent ALI differentiation^39^. Therefore, we cannot rule out the possibility that several small molecules, such as the Notch inhibitor DAPT which promotes basal cell expansion^40,41^, differentially affect the mucociliary differentiation of nasal and bronchial cells in ALI cultures. Furthermore, multiple studies have demonstrated that the differentiation medium applied in ALI cultures can also influence airway epithelial cell composition and functioning^42–44^. This could potentially explain why others, including a recent single-cell RNA sequencing study^45^, have not observed differences in mucociliary cell composition between ALI-differentiated nasal and bronchial airway epithelial cells. Therefore, further studies are required to gain deeper insight into how intrinsic differences between nasal and bronchial epithelial cells persist under different expansion and differentiation culture conditions.

In addition to differences in mucociliary differentiation, we also observed a lower barrier integrity in nasal cell cultures compared to bronchial cells. This correlated with differences in mRNA expression of several adherens and tight junctions-related genes. It can be speculated that this reduced expression and the resulting decrease in TEER could be linked to the enhanced presence of goblet cells in nasal cell cultures. This corresponds with previous studies that examined the effect of IL-13 on bronchial epithelial cells. In addition to inducing goblet cell differentiation, IL-13 has been demonstrated to compromise the airway epithelial barrier and downregulate the expression of specific claudins^46,47^. However, further studies are needed to gain a deeper understanding of the factors contributing to the differences in barrier integrity between nasal and bronchial epithelial cell cultures.

Following the insights obtained from our RNA seq data in ALI cultures, IF analysis revealed the presence of key TF proteins related to nasal (PAX6 and SIX3) and bronchial (NKX2-1 and FOXA2) epithelial cells already in basal progenitor cells. Moreover, using ATAC-seq we have demonstrated that multiple key TF genes and their putative binding sites are associated with accessible chromatin specifically in nasal or bronchial progenitor cells. These findings support the hypothesis that the high abundance of goblet cells in nasal cell cultures is due to the absence of certain transcriptional regulatory proteins that are expressed in bronchial epithelial cells. For instance, the TFs FOXA1/2 and NKX2-1, which are not expressed in nasal cells, are known for their inhibitory effect on goblet cell metaplasia in bronchial epithelial cells^20,48,49^. Besides FOXA1/2, nasal cells lacked both mRNA expression in ALI-cultures and accessible TF DNA binding motifs in basal cell cultures of other regulatory proteins that have been previously reported to regulate endoderm-derived lung development, such as IRX1-3^22,23^. In contrast, nasal cell-enriched TFs included PAX6, FOXG1, OTX2, and SIX3, which have been reported to regulate the development of ectoderm-derived tissues^24,29,30^. This suggests that unique nasal- and bronchial-specific TFs are likely related to differences in their germ layer origin. Further research is required to demonstrate the role of nasal cell-specific TFs in both nasal epithelial development and the regulation of mucociliary differentiation. For instance, as shown in ectoderm-derived epithelial tissues^50,51^, PAX6 may act as key regulator of nasal epithelial cell differentiation. It will be interesting for subsequent studies to characterize the role of these nasal cell-specific TFs, for instance by gene knockout or overexpression studies, as they likely drive tissue-specific differences in upper and lower respiratory disease development—both in CF and other respiratory diseases.

Our results further revealed clear differences in nasal and bronchial organoid fluid secretion. In earlier studies we showed that nasal organoids from subjects with CF displayed high intrinsic and cAMP-induced CFTR-independent fluid secretion^13,18^. In the current study we observed that bronchial organoids of subjects with CF lacked CFTR-independent fluid secretion. In contrast to CF nasal organoids, bronchial organoids responded to CFTR-modulating drugs, further suggesting differences in CFTR-dependent fluid secretion. It should be mentioned that the effect of CFTR modulator responses was measured in a limited number of available donors. Furthermore, we showed previously that adaption of the culturing conditions of CF nasal organoids is needed to boost CFTR expression and make them suitable for CFTR-dependent FIS-assays^18^. Therefore, the observed effects of CFTR modulators in nasal and bronchial organoids should be validated in future efforts using a larger cohort and optimized organoid culture conditions. Of note, in contrast to our observations in nasal and bronchial organoids, others did not observe differences in CFTR-dependent chloride conductance between ALI-differentiated nasal and bronchial epithelial cells^52,53^. More research is needed to gain insight into the mechanisms underlying the differential CFTR-independent fluid secretion between nasal and bronchial organoids, which may relate to the differential expression of ion channel and transporters between nasal and bronchial cells, as observed in our RNA seq analysis.

In an attempt to link the differences in fluid secretion to altered cell differentiation, we used the Notch inhibitor DAPT to enrich for ciliated cells in nasal organoids. While this indeed reduced intrinsic fluid secretion, we did not observe changes in FIS, suggesting different mechanisms underlying the intrinsic and cAMP-induced CFTR-independent fluid secretion. Moreover, differentiation with DAPT did not improve CFTR modulator responses, which is likely due to a depletion of CFTR-expressing secretory cells^54,55^. Overall, our data suggest that epithelial cell differentiation is an important determinant of CFTR-independent epithelial fluid secretion. Additionally, it provides a proof-of-concept that differentiation studies in ALI-cultures can be combined with the assessment of fluid secretion in ALI-culture derived organoids.

Together, our data show that nasal and bronchial epithelia have unique phenotypic and functional characteristics that persist in cell culture, including intrinsic differences in germ layer-specific gene regulatory networks and cellular differentiation, which may affect epithelial fluid secretion in CF. Cultured nasal and bronchial epithelial cells may therefore serve as excellent models to further explore the contribution of tissue-specific characteristics on upper and lower respiratory disease development in CF.

## Materials and methods

### Patient materials and sample collection

Paired nasal and bronchial samples were collected as part of the Precision study (protocol ID: NL54885.041.16), which was approved by the Medical Research Ethics Committee of the University Medical Center Utrecht (Utrecht, The Netherlands). Paired samples were collected from 1 child without CF (Female (F)) and 8 children with CF with the following *CFTR* mutations: F508del/F508del (F), F508del/F508del (F), F508del/F508del (F), F508del/F508del (F), F508del/A455E (Male (M)), F508del/A455E (F), F508del/1717-1G>A (M), F508del/2183AA>G (F). Additionally, nasal brushings were obtained from subjects that gave signed informed consent for use and storage of their cells, which was approved by a specific ethical board for the use of biobanked materials TcBIO (Toetsingscommissie Biobanks), an institutional Medical Research Ethics Committee of the University Medical Center Utrecht (protocol ID: 16/586). These non-paired nasal brushings were obtained from 1 child with CF (F508del/F508del (F)), 3 adults with CF (all F508del/F508del (M)) and 7 adult healthy controls. Furthermore, residual bronchial tissues from lung transplantation donors at the University Medical Center Utrecht, the Netherlands, were accessible for research within the framework of patient care, in accordance with the “Human Tissue and Medical Research: Code of conduct for responsible use” (2011) (http://www.federa.org), describing the no-objection system for coded anonymous further use of such tissue without necessary written or verbal consent. These bronchial tissues were obtained from 3 subjects without CF. All nasal samples were obtained as brushings from both inferior turbinates by use of a cytological brush. Bronchial samples from the CF subjects were obtained as brushings during a bronchoscopy and from the healthy controls as explant material. All samples were collected in advanced DMEM/F12 containing glutaMAX (1% v/v), HEPES (10 mM), penicillin–streptomycin (1% v/v) and primocin (50 mg/mL). Participants’ ages ranged from 1 to 29 years (mean = 8.72, SD = 9.8) with 73% identified as female, 27% as male and all having the Dutch nationality.

### Isolation and expansion of airway epithelial cells

Nasal and bronchial airway epithelial cells were isolated from nasal and bronchial brushings as previously described^18^. In brief, cells were scraped off the brush, incubated with TrypLE express enzyme (Fisher Scientific, Landsmeer, The Netherlands) supplemented with sputolysin for 10 min at 37 °C, strained with a 100 µM strainer and plated in a collagen IV-precoated (50 µg/mL) 6-well culturing plate. Cells were refreshed three times a week with basal cell (BC) isolation medium (Supplementary Table S3). After one week, antibiotics were withdrawn from the medium and DAPT was added to the medium, which was called BC expansion medium (Supplementary Table S3) from now on. These basal progenitor cells were cultured until 80–90% confluence. Confluent cell layers were frozen in CryoStor CS10 freezer medium (STEMCELL technologies, Vancouver, Canada) supplemented with Y-27632 (5 µM; Selleck chemicals, Planegg, Germany), or passaged using TryplE express enzyme. Bronchial airway epithelia were isolated from explant tissue by first incubating resected bronchial tissues in Protease type XIV (Sigma-Aldrich, St Louis, MO, USA) at 4 °C overnight. The next day, dissociated epithelial cells were treated with TrypLE express enzyme (Thermo Fischer Scientific, Waltham, MA, USA) to obtain single cells, which were isolated and expanded in 2D cell cultures, using similar culture conditions as the nasal epithelial cells.

### ALI differentiation of airway epithelial cells

Mucociliary differentiation of nasal and bronchial airway epithelial cells (passage = 3 or 4) was conducted in ALI-Transwell cultures as previously described^18^. Similar passages were used in direct comparisons of paired nasal and bronchial epithelial cells. In brief, 0.2 × 10^6^ or 0.5 × 10^6^ basal progenitor cells (24 or 12 well-inserts respectively) were seeded on PureCol-coated (30 μg/mL, Advanced BioMatrix, Carlsbad, CA, USA) Transwell inserts (0.4 μm pore size polyester membrane, Corning, Corning, NY, USA). Cells were first cultured in submerged conditions with BC expansion medium (Supplementary Table S3) until 100% confluence. Medium was then changed to ALI differentiation medium (Supplementary Table S4) supplemented with A83-01 (500 nM). After 2 days, apical medium was removed to culture the cells under air-exposed conditions. A83-01 was withdrawn from the medium after 3–4 days at air-exposed conditions. Medium was refreshed twice a week, and the apical side of the cells was washed with PBS once a week. Cells were differentiated for 18 days at air-exposed conditions. In indicated experiments, DAPT (20 µM) was added to ALI-differentiation medium from the 4th day of air-exposure.

### Conversion of ALI-differentiated airway epithelia into 3D organoids

Differentiated ALI-cultures were converted into 3D airway organoids, as previously described^18^. In brief, ALI-cultures were treated with collagenase type II (1 mg/mL, Thermo Fisher Scientific) for 45–60 min at 37 °C to detach the epithelial layer from the Transwell membrane. After detachment, the epithelial layer was mechanically disrupted into fragments by pipetting and subsequently strained with a 100 µM strainer. After centrifugation, epithelial fragments were resuspended in ice-cold 75% Matrigel (Corning, v/v in airway organoid medium) and plated as 30 µL droplets on pre-warmed 24-well suspension plates. These plates were then placed upside down in a tissue incubator for 20–30 min to solidify the Matrigel droplets. Airway organoid medium was added and refreshed twice a week to stimulate airway organoid formation (Supplementary Table S5). It takes 1–3 days for organoid formation and the development of intrinsic lumen. In indicated experiments, DAPT (20 µM) was added to the airway organoid medium.

### Forskolin-induced swelling (FIS) measurements in airway organoids

One or two days before fluid secretion measurements, airway organoids were transferred to 96-well plates in 4 µL droplets of 75% Matrigel (v/v in airway organoid medium). Organoids were cultured with 100 µL airway organoid medium. For a FIS assay, airway organoids were stained with calcein green AM (3 µM, Invitrogen, Waltham, MA, USA) 30 min before the experiment. Airway organoids were then stimulated with forskolin (5 µM) and organoid swelling was visualized by imaging at 15-min time intervals for a period of 2 h. Images were made with a Zeiss LSM800 confocal microscope (Zeiss, Breda, Netherlands) at 37 °C and 95% O_2_/5% CO_2_, using a 5 × objective. To determine CFTR modulator responses, CF airway organoids were pre-incubated with the CFTR corrector VX-809 (10 µM, Selleck chemicals, Planegg, Germany) for 48 h, followed by stimulation with the CFTR potentiator VX-770 (10 µM, Selleck Chemicals, Planegg, Germany) together with forskolin (5 µM, Sigma-Aldrich, St Louis, MO, USA). Total organoid area per image was quantified using Zen Blue Software (Zeiss). Organoid swelling was then calculated over time, normalized for t = 0 and the baseline was set at 100%. Organoid swelling was also expressed as area under the curve (AUC) values to better compare different conditions. Organoid swelling experiments were performed in quadruplicates.

### Organoid size measurements

Organoid size was quantified by use of the OrgaQuant convolutional neural network which automatically recognizes organoids in brightfield images^13,56^. The organoid surface area was estimated using OrgaQuant bounding boxes, assuming organoids had a disk shape. The mean surface area of all individual organoids within a culture well was used for further analyses.

### Immunocytochemistry

Undifferentiated airway epithelial cells cultured at collagen IV-coated ibidi 18-well slides, ALI-differentiated airway epithelia, and organoids plated in a 96-well plate, were stained as previously described^12,18,57^, using indicated antibodies (Supplementary Table S6), with or without phalloidin and DAPI. Images were acquired with a Leica SP8X confocal microscope or Leica THUNDER imager and processed with LAS X software and ImageJ/FIJI. For quantification, MUC5AC and β-tubulin IV staining was analyzed in three microscopic fields per well. Relative fluorescence was quantified by calculating the area of the image occupied by MUC5AC- or β-tubulin IV staining above a specific threshold, and expressed as arbitrary units. Microscope settings and thresholds were similar for all images.

### Quantitative real time PCR

Total RNA was extracted from ALI-differentiated nasal and bronchial airway epithelial cells using the RNeasy Mini Kit (Qiagen, Venlo, Netherlands) according to the manufacturer’s protocol. For quantitative real-time PCR (qPCR), cDNA was first synthesized with the iScript cDNA synthesis kit (Bio-Rad, Hercules, CA, USA) according to the manufacturer’s protocol. qPCR was conducted using mixtures of specific primer pairs (Supplementary Table S7) and iQ SYBR Green Supermix (Bio-Rad), using a CFX96 real-time detection machine (Bio-Rad). CFX Manager 3.1 software (Bio-Rad) was used to calculate relative gene expression normalized to the housekeeping genes *ATP5B* and *RPL13A* according to the standard curve method. The housekeeping genes were selected based on stable expression in airway epithelial cells using the “Genorm method”^58^. Experiments were performed with two technical replicates.

### RNA-seq

RNA-seq was performed at Single Cell Discoveries, using an adapted version of the CEL-seq protocol^59,60^. Total RNA concentration was measured and normalized to 20 ng/µL using a Qubit fluorometer (Invitrogen, Waltham, MA, USA), and RNA quality was assessed via bioanalyzer and RNA Pico 6000 kit (Agilent, Santa Clara, CA, USA). Normalized total RNA (with RNA integrity number (RIN) scores > 7) was used for library preparation and sequencing. Samples were barcoded with CEL-seq primers during a reverse transcription and pooled after second strand synthesis. The resulting cDNA was amplified with an overnight in vitro transcription reaction. From this amplified RNA, sequencing libraries were prepared with Illumina Truseq small RNA primers. The DNA library was paired-end sequenced on an Illumina Nextseq™ 500, high output, with a 1 × 75 bp Illumina kit (R1: 26 cycles, index read: 6 cycles, R2: 60 cycles), and a sequencing depth of 12 million reads per sample. Read 1 was used to identify the Illumina library index and to check for valid CEL-seq sample barcodes that have a maximum Hamming distance of 1. Read 2 was trimmed and aligned to the human GRCh38 reference transcriptome using BWA (version 0.7.15) MEM -t 8 and other parameters were set to default^61^. Reads that mapped equally well to multiple locations were discarded. Mapping and generation of count tables was automated and carried out using the MapAndGo (version 3.0) script^62^. The number of raw input reads was 195.028.544 and 92% of these had a valid sample barcode. 15.771.259 of the raw reads had an incorrect cell sample barcode. After mapping, 131.632.003 reads mapped to the transcriptome with a mappability of 73%. Differential gene expression analysis was performed using the R package DEseq2 (version 1.30.1)^63^. Correction for donor and LFC shrinkage was performed using apeglm (version 1.12.0)^64^. A gene was considered differentially expressed when the adjusted p value was < 0.01 and fold change > 1.5 or < − 1.5. Gene set enrichment analysis for gene ontology of biological processes was performed using clusterProfiler (version 3.18.1)^65^ and visualized using enrichplot (version 1.10.2)^66^.

### Initial processing of ATAC-seq data, peak calling and peak-gene assignment

ATAC-seq reads were aligned to the human genome (hg38) with HISAT2 using the Octopus Toolkit^67^. HOMER’s^68^ makeTagDirectory command was used to make Tag Directories, which were manually filtered to remove reads mapping to the Y chromosome or mitochondrial genome. The fragment length estimate was set to 64 as a representative estimate for all samples. The average signal at all transcription start sites (TSS) in the genome, and the local background signal surrounding these TSS, were calculated using HOMER’s annotatePeaks command (options tss hg38 -size 10,000 -hist 40). The relative quality of each individual sample was determined based on its specific TSS enrichment ratio, which was defined as the average TSS signal (− 80 to + 80 bp from TSS) divided by the average local background signal (− 5 kb to − 4.96 kb and + 4.96 kb to + 5 kb away from TSS). Peak calling was performed using HOMER’s findPeaks command (options -region -size 100 -minDist 75 -localSize 50,000). HOMER’s mergePeaks command was used to identify peaks that were present in at least 3/4 (for bronchial samples) or 3/5 (for nasal samples) biological replicates. Only these ‘reproducible peaks’ were kept for downstream analysis. Peaks that were present in all samples (‘universal peaks’) were identified as overlapping reproducible peaks found in both experimental groups. HOMER’s makeUCSCfile command was used to create bedGraph files for data visualization of in the IGV genome browser. Peaks were assigned to putative target genes using GREAT with default settings (i.e. Basal plus extension; proximal: 5 kb upstream, 1 kb downstream, distal: up to 1000 kb)^69^.

### Downstream analysis of ATAC-seq data

Raw counts were determined at all reproducible peaks using HOMER’s annotatePeaks command (options hg38 -size given -raw)^68^. Counts were normalized using the R package DESeq2^63^. To accommodate for the relative lack of zero’s in ATAC-seq data at regions without true signal, we slightly modified DESeq2’s standard normalization method. Briefly, scaling factors were determined for each sample based on the set of universal peaks (i.e. peaks that were present in all samples) and were used to normalize counts at all peaks. PCA of all reproducible peaks (n = 15,434) was performed with FactoMineR^70^. Differentially enriched ATAC-Seq peaks were identified using DESeq2 (log2 fold change > 1, adjusted p value < 0.1), with scaling factors based on the universal peak set. Pathway enrichment analysis of genes near bronchial-specific and nasal-specific ATAC-seq peaks was performed using Metascape^71^. Bronchial-specific and nasal-specific ATAC-seq peaks were used as input for HOMER’s findMotifsGenome script (options -size 200 -mask -len 6,8,10,12 -S 20) to search for known transcription factor binding motifs.

### Statistical analysis

Statistical analyses were performed using Graphpad version 9.3.0 or R version 4.0.3. Data is presented as mean ± SD, unless otherwise indicated. Statistical tests used for analysis of differences are indicated in corresponding figure legends. Differences were considered significant at p < 0.05.

### Ethics declarations

The study was conducted according to the guidelines of the Declaration of Helsinki, and approved by the Medical Research Ethics Committee of the University Medical Center Utrecht (Utrecht, The Netherlands) (protocol ID: NL54885.041.16, approved on 16 November 2016 and protocol ID: 16/586, approved on 25 January 2017). Informed consent was obtained from all subjects involved in the study.

### Supplementary Information

Supplementary Figure S1.

Supplementary Figure S2.

Supplementary Figure S3.

Supplementary Legends.

Supplementary Table S1.

Supplementary Table S2.

Supplementary Table S3.

Supplementary Table S4.

Supplementary Table S5.

Supplementary Table S6.

Supplementary Table S7.

## Data Availability

Normalized counts of sequencing can be found in the supplementary tables. Deidentified raw counts from sequencing data have been deposited in the NCBI Gene Expression Omnibus database under accession number GSE241801. Raw sequencing data cannot be provided with the manuscript due to privacy concerns of the human subjects.
