# Supplementary figures and images for: Exploring intrinsic variability between cultured nasal and bronchial epithelia in cystic fibrosis
Source: Sci Rep. 2023 Oct 30;13:18573. doi: 10.1038/s41598-023-45201-4 (PMC10616285; doi:10.1038/s41598-023-45201-4)

Supplementary Figure 1

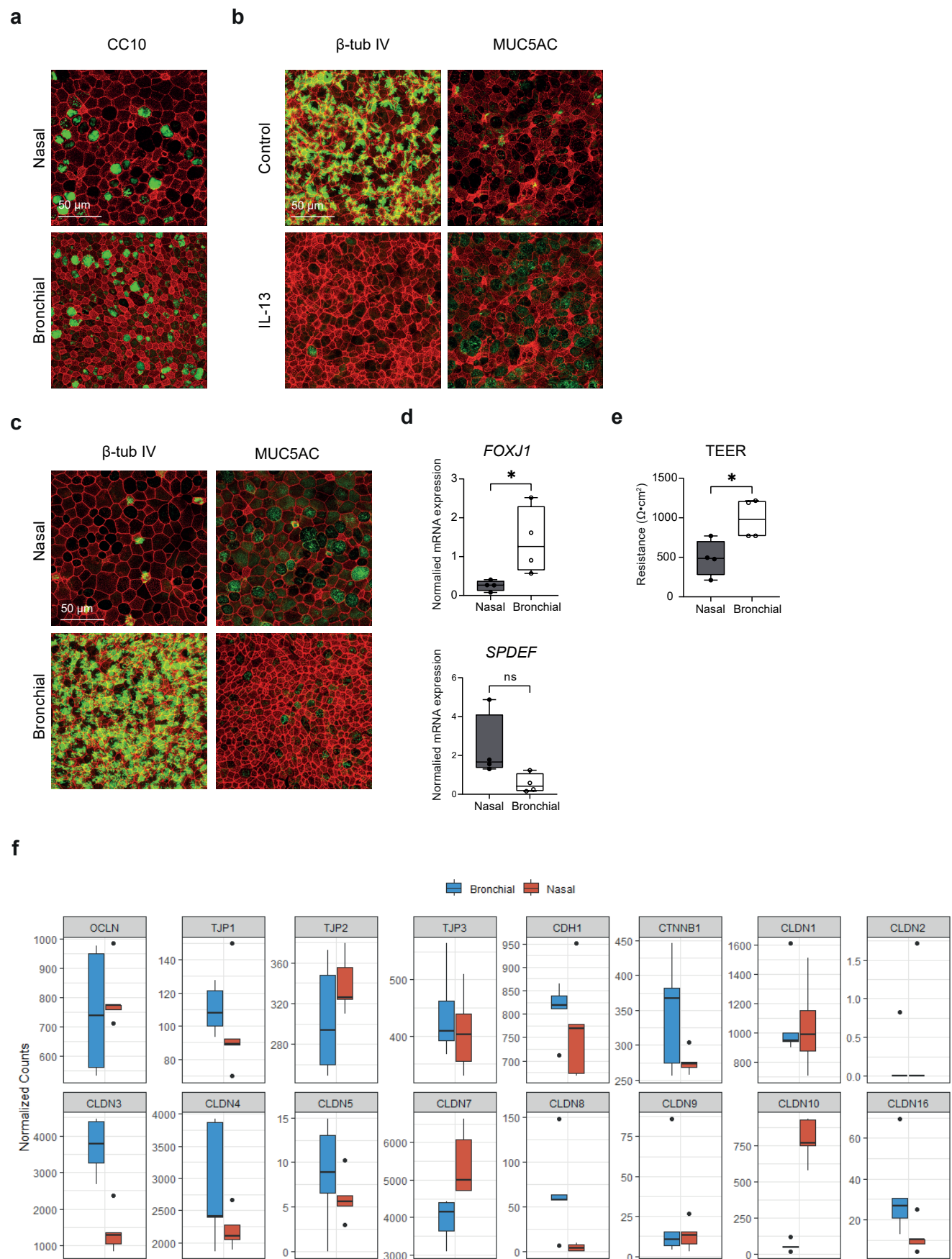

Supplement: Supplementary file 1 — Supplementary Figure S1. [file 41598_2023_45201_MOESM1_ESM.pdf]

Supplementary Figure 2

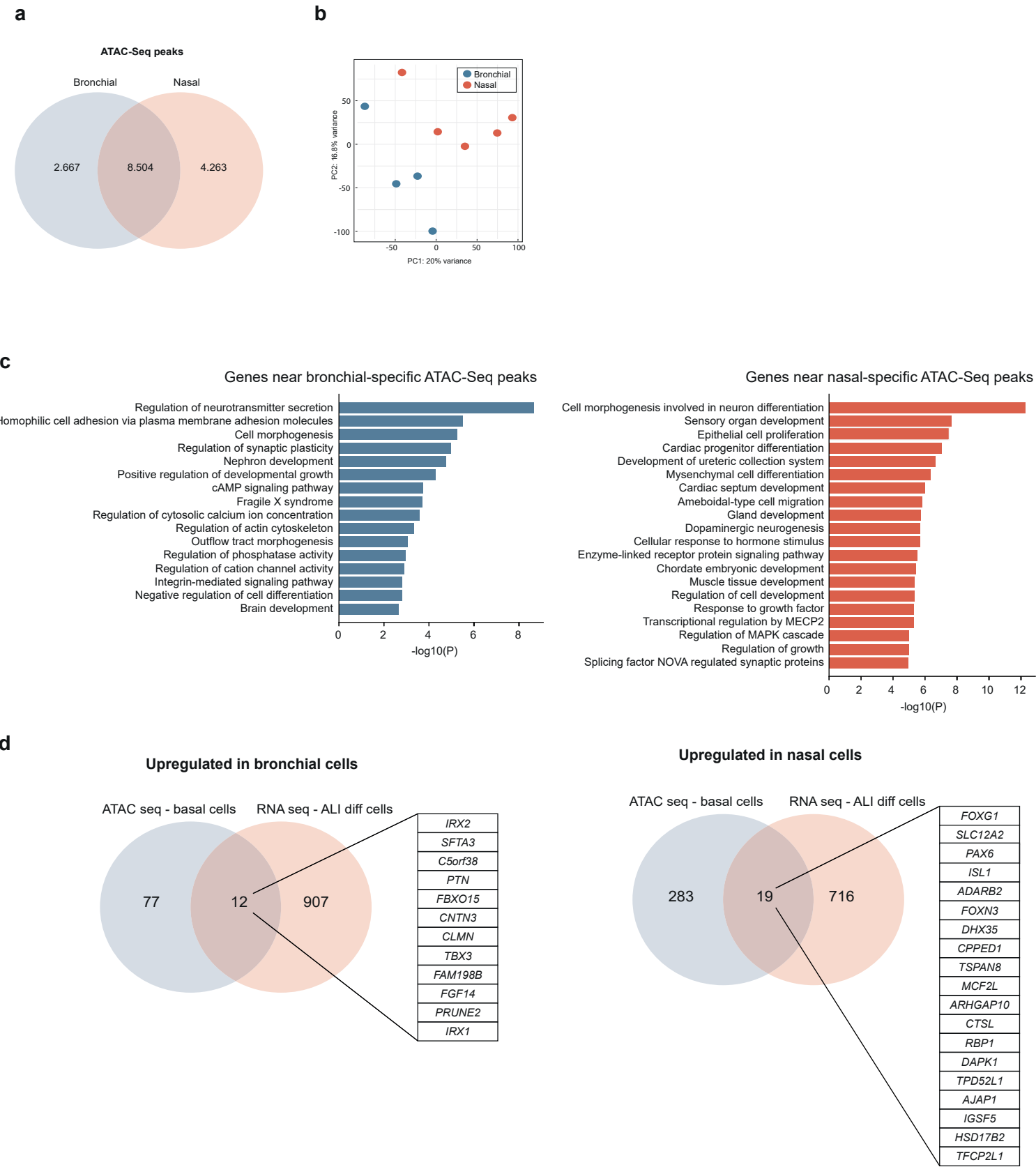

Supplement: Supplementary file 2 — Supplementary Figure S2. [file 41598_2023_45201_MOESM2_ESM.pdf]

# Supplementary Figure 3

a

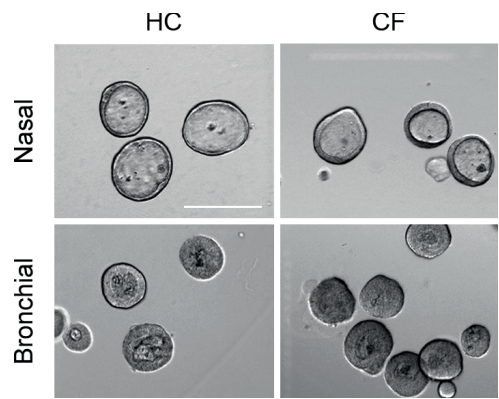

b

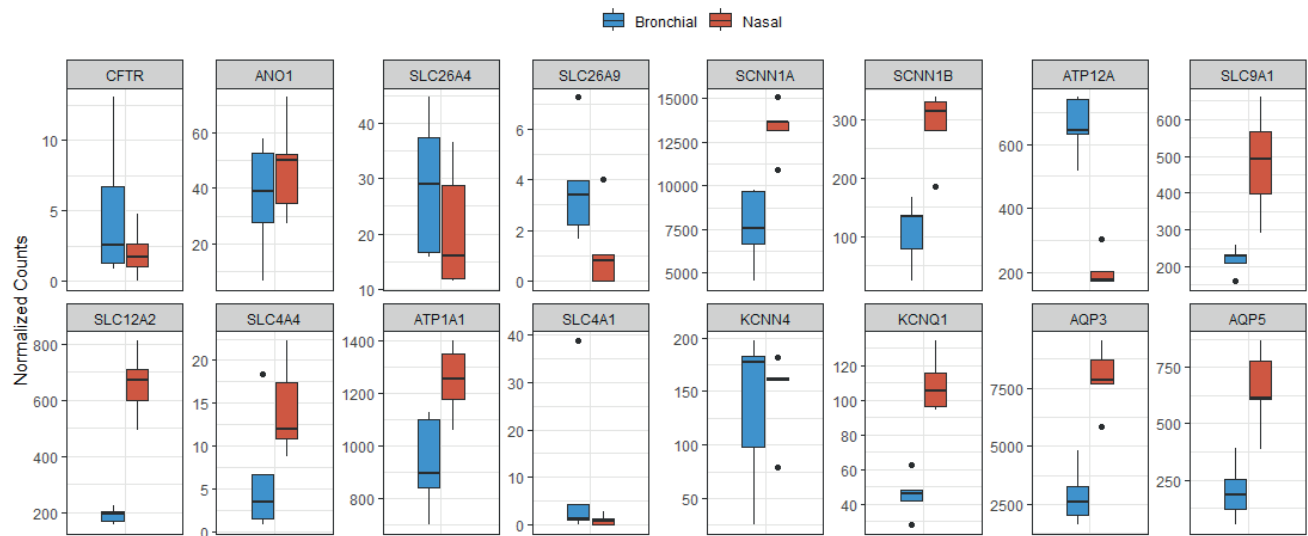

c

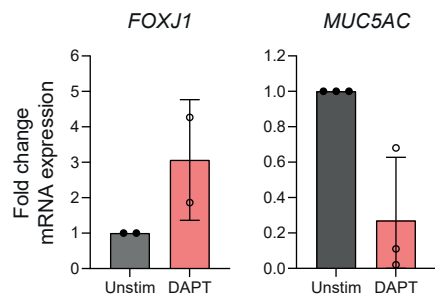

d

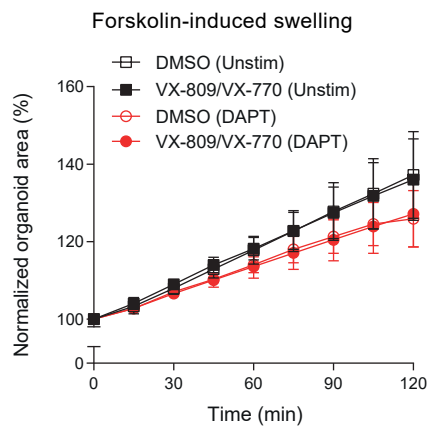

Supplement: Supplementary file 3 — Supplementary Figure S3. [file 41598_2023_45201_MOESM3_ESM.pdf]
