# Supplementary material for: Exploring intrinsic variability between cultured nasal and bronchial epithelia in cystic fibrosis
Source: Sci Rep. 2023 Oct 30;13:18573. doi: 10.1038/s41598-023-45201-4 (PMC10616285; doi:10.1038/s41598-023-45201-4)
Supplement: Supplementary file 4 — Supplementary Legends. [file 41598_2023_45201_MOESM4_ESM.pdf]

Exploring intrinsic variability between cultured nasal and bronchial epithelia in cystic fibrosis

Lisa W. Rodenburg[1,2*], Mieke Metzemaekers[3,4], Isabelle S. van der Windt[1,2], Shannon M.A. Smits[1,2], Loes A. den Hertog – Oosterhoff[1,2], Evelien Kruisselbrink[1,2], Jesse E. Brunsveld[1,2], Sabine Michel[1], Karin M. de Winter-de Groot[1], Cornelis K. van der Ent[1], Ralph Stadhouders[3,4], Jeffrey M. Beekman[1,2,5], Gimano D. Amatngalim[1,2]

Supplementary Information

Supplementary Table S1: Differentially expressed genes between ALI-differentiated nasal and bronchial epithelial cells

Supplementary Table S2: Differentially enriched ATAC-seq peaks between nasal and bronchial basal progenitor cells

Supplementary Table S3: BC isolation and BC expansion medium

Supplementary Table S4: ALI-differentiation medium

Supplementary Table S5: Airway organoid medium

Supplementary Table S6: Antibodies for immunofluorescence

Supplementary Table S7: Primers for qPCR

Supplementary Figures S1-S3

Supplementary Figure S1) Nasal and bronchial epithelial cell cultures exhibit unique mucociliary differentiation states

a) Representative IF staining of CC10 (club(-like) cells) in paired ALI-differentiated CF nasal and bronchial epithelial cells (F508del/F508del). Cultures were differentiated for 18 days. Epithelial markers are shown in green, phalloidin (red) was used as actin cytoskeleton staining. Scale bar equals 50 µm.

b) Representative IF staining of β-tubulin IV (ciliated cells) and MUC5AC (goblet cells) bronchial cells differentiated in ALI-cultures at control conditions or treatment with IL-13 (10 ng/ml) for 18 days. . Epithelial markers are shown in green, phalloidin (red) was used as actin cytoskeleton staining. Scale bar equals 50 µm.

c) Representative IF staining of β-tubulin IV and MUC5AC in ALI-differentiated nasal and bronchial cells of a HC subject. Cultures were differentiated for 18 days. Epithelial markers are shown in green, phalloidin (red) was used as actin cytoskeleton staining. Scale bar equals 50 µm.

d) mRNA expression of the cell type-specific transcriptional factors *FOXJ1* (ciliated cells) and *SPDEF* (goblet cells) in ALI-differentiated nasal (grey bars) and bronchial (open bars) epithelial cells of HC subjects (n=4 independent donors).

e) TEER measurements of ALI-differentiated nasal (grey bars) and bronchial (open bars) epithelial cells of HC subjects (n=4 independent donors).

   Data is shown as mean ± SD. Analysis of differences was conducted using unpaired t-tests (panel b, c). ns = non-significant, * $p<0.05$.

f) Normalized counts of adherens and tight junction associated genes, including occludin (OCLN), ZO-1-3 (TJ1-3), E-cadherin (CDH1), β-catenin (CTNNB1), and claudins (CLDN1-16) in paired ALI-differentiated nasal (red) and bronchial (blue) cells of CF

subjects (n=5 independent donors; F508del/F508del, F508del/F508del, F508del/A455E, F508del/A455E, F508del/1717-1G>A).

Supplementary Figure S2) Unique epigenomic features of nasal and bronchial basal progenitor cells

a) Venn diagram showing the identified ATAC-seq peaks, indicating chromatin accessible regions, and discriminates between nasal- and bronchial-specific ATAC-seq peaks.

b) PCA of differentially enriched ATAC-seq peaks in undifferentiated nasal (n=5 donors, (4 donors carrying the F508del/F508del mutation, 1 donor F508del/A455E), of which 4 paired donors) and bronchial epithelial cells (n = 4 paired donors) (log2 fold change >1, adjusted $p < 0.1$).

c) Pathway enrichment analysis with genes assigned to the bronchial- (left panel) and nasal- (right panel) specific differentially expressed ATAC-seq peaks.

d) Venn diagram showing overlap between genes corresponding to differentially expressed ATAC-seq peaks in undifferentiated bronchial (left panel) and nasal (right panel) epithelial cells and DEGs from RNA-seq, enriched in ALI-differentiated bronchial epithelial cells.

Supplementary Figure S3) ALI culture-derived nasal and bronchial organoids display distinctive fluid secretion, which is dependent on the amount of ciliated cells

a) Representative brightfield images of nasal and bronchial airway organoids from HC and CF subjects (F508del/2183AA>G), demonstrating pre-swollen lumens in HC and CF nasal organoids, and a lack of lumen formation in bronchial organoids. Scale bar equals 500 µm.

b) Normalized counts of ion channels and transporters, including apical *expressed CFTR, ANO1, SLC26A4, SLC26A9, SCNN1A, SCNN1B* (both ENaC subunits), and *ATP12A,*

basolateral expressed NBC (*SLC4A4*), NKCC1 (*SLC12A2*), the ATPase Na+/K+ transporting subunit alpha 1 (*ATP1A1*), and K+ channels (*KCNJ15* and *KCNQ1*) in paired ALI-differentiated nasal (red) and bronchial (blue) cells of CF subjects (n=5 independent donors; F508del/F508del, F508del/F508del, F508del/A455E, F508del/A455E, F508del/1717-1G>A).

c) mRNA expression of the cell type-specific transcription factors *FOXJ1* (ciliated cells) and *MUC5AC* (goblet cells) in nasal cells from individuals with CF (n=2-3 independent donors; F508del/F508del, F508del/F508del, F508del/1717-1G>A), treated with (red) or without (grey) the γ-secretase inhibitor DAPT (20 µM). mRNA expression is calculated using the ddCt method and shown as a fold change compared to unstimulated cells.

d) Nasal organoid swelling assay with organoids from individuals with CF (n=3 independent donors; al; F508del/F508del), treated with or without the γ-secretase inhibitor DAPT (20 µM). Organoids were stimulated with forskolin (5 µM) alone, or with forskolin (5 µM) together with VX-770 (10 µM) and pre-treatment with VX-809 (10 µM) for 48 h. Results are depicted as percentage increase in normalized area in time. Data is shown as mean ± SD. Analysis of differences was conducted with a one-sample t-test (panel b).
